# Nature-inspired visual interventions in metro stations and commuters’ affective well-being: the mediating roles of perceived crowding and environmental comfort

**DOI:** 10.3389/fpsyg.2026.1901802

**Published:** 2026-08-04

**Authors:** Weixin Zhu, Yi Yu

**Affiliations:** College of Arts and Design, Ningbo University of Finance and Economics, Ningbo, Zhejiang, China

**Keywords:** affective well-being, environmental comfort, metro station, nature-inspired visual intervention, perceived crowding, restorative visual design

## Abstract

**Objective:**

This study examined whether restrained nature-inspired visual interventions in metro stations improve commuters’ momentary affective well-being and whether perceived crowding and environmental comfort explain.

**Methods:**

An image-based between-subjects experiment was conducted with 360 adult metro commuters, who were randomly assigned to conventional station scenes or nature-inspired intervention scenes. The intervention added coherent nature-inspired imagery and low-saturation green/blue-green tones to existing display and wall-surface areas while keeping spatial layout, passenger number and arrangement, lighting, signage, viewing perspective, and facilities constant. After viewing the assigned scenes, participants rated perceived visual quality, perceived crowding, environmental comfort, and momentary affective well-being. Independent-samples *t*-tests and serial mediation analysis were used to test the hypotheses.

**Results:**

Compared with the conventional condition, the nature-inspired intervention condition produced lower perceived crowding, higher environmental comfort, and higher momentary affective well-being. Perceived crowding and environmental comfort each mediated the relationship between visual condition and affective well-being. The serial pathway was also significant: the intervention reduced perceived crowding, which was associated with greater environmental comfort and, in turn, better affective well-being. After the mediators were included, the direct effect of experimental condition was no longer significant.

**Conclusion:**

Restrained nature-inspired visual interventions can improve commuters’ affective experience in metro stations by shaping appraisals of crowding and comfort. These findings extend the metro transit S-O-R model by showing how a targeted restorative visual strategy may ease perceived spatial pressure and enhance momentary well-being in high-density public transport settings. The results should be interpreted as evidence for the tested visual strategy rather than for all forms of station design improvement.

## 1 Introduction

Metro stations are important everyday public transport spaces in which passengers wait, walk, transfer, and enter or leave the transport system. Their visible environmental elements include colors, wall surfaces, digital displays, signs, decorative images, and the overall visual organization of the station environment. In environmental psychology and urban design research, perceived visual quality is commonly associated with aesthetic appeal, visual harmony, orderliness, imageability, human scale, and psychologically supportive characteristics (Nasar, 1994; Ewing and Handy, 2009; Ríos-Rodríguez et al., 2021; Miola and Pazzaglia, 2025). However, visual environmental quality is a broad and multidimensional concept rather than a single design attribute.

It is therefore important to clarify the scope of the present visual intervention at the outset. This study does not examine overall metro visual environmental quality in a broad sense. It does not test lighting design, material texture, dynamic advertising screens, signage layout, information hierarchy, spatial configuration, architectural renovation, or comprehensive station branding. Rather, it focuses on a bounded and practically feasible intervention: coherent nature-inspired digital imagery and low-saturation green or blue-green tones applied to existing display and wall-surface areas. The conclusions should therefore be interpreted as evidence for this restrained restorative visual strategy, not as evidence that all forms of visual enrichment or all station design upgrades will improve commuters’ affective experience.

The literature relevant to this study can be organized into three connected research threads. The first thread concerns restorative environments, nature exposure, and biophilic visual design. Attention Restoration Theory and Stress Recovery Theory suggest that natural or nature-like environments can support attentional recovery, reduce stress, and promote positive affective states (Kaplan, 1995; Ulrich et al., 1991; Hartig et al., 2003; Berto, 2005, 2014). More recent work further indicates that nature exposure and biophilic indoor environments may contribute to mental health, autonomic recovery, and stress or anxiety reduction (Brown et al., 2013; Bratman et al., 2019; Yin et al., 2020). These studies provide the theoretical basis for examining whether restrained nature-inspired imagery may have restorative value even in highly functional underground transit spaces. Meta-analytic and systematic-review evidence further suggests that contact with natural environments can improve affective states and that the attention-restoration potential of nature exposure has empirical support, although effects vary across outcome measures and exposure formats (McMahan and Estes, 2015; Ohly et al., 2016).

The second thread concerns commuting psychology, perceived crowding, comfort, and travel well-being. Commuting is not only a utilitarian mobility activity but also a repeated affective experience. Travel satisfaction is related to subjective well-being and emotional well-being, while public transport users may experience waiting, limited personal space, crowding, and environmental discomfort (Bergstad et al., 2011; Ettema et al., 2011, 2012; De Vos et al., 2013; Friman et al., 2017; Olsson et al., 2013). Within this literature, perceived crowding is distinguished from objective passenger density and is known to influence stress, exhaustion, comfort, route choice, and public-transport valuation (Stokols, 1972; Evans and Wener, 2007; Mohd Mahudin et al., 2012; Li and Hensher, 2011; Tirachini et al., 2013; Lombardi and Ciceri, 2021; Yap et al., 2020). This thread suggests that a visual intervention may matter not only by improving aesthetic appraisal but also by altering how the same level of passenger presence is psychologically experienced. Broader meta-analytic work on perceived crowding also shows that human crowding and spatial crowding can operate differently and that crowding influences emotional responses partly through perceived control and situational appraisal, which supports the present distinction between objective density and subjective crowding (Blut and Iyer, 2020).

The third thread concerns metro station design and passenger experience in Chinese and East Asian transit contexts. Studies have increasingly treated station design as a passenger-experience and environmental-psychology issue rather than a purely engineering matter. Hu et al. (2019) argue that subway station space should be optimized in relation to accessibility, convenience, comfort, and cultural experience. Hu and Xu (2023), using a Nanjing metro virtual-reality experiment, show that signage configuration can affect environmental pressure and wayfinding performance. Su and Ding (2024) summarize Chinese subway public-space design research and emphasize human-centered, green, cultural, and cross-disciplinary approaches. Related work has examined subway station comfort through EEG field measurements, indoor greenness through psychophysiological responses, and environmental characteristics associated with emotional perception (Kim et al., 2020; Kim and Lee, 2021; Shi et al., 2025). These studies show that metro station experience is increasingly studied in relation to psychological responses, but they have less often tested a controlled restorative visual intervention together with a serial mediation pathway linking visual condition, perceived crowding, environmental comfort, and affective well-being.

Against these three research threads, the present study aims to make a specific contribution. It does not simply ask whether a station looks better after visual renovation. Instead, it tests whether a restrained nature-inspired visual intervention can influence commuters’ momentary affective well-being through two theoretically specified appraisals: perceived crowding and visually induced environmental comfort. This focus helps distinguish the present work from broader studies of metro spatial design, signage optimization, cultural decoration, or general station environmental quality.

At the same time, restorative visual effects should not be assumed for all kinds of visual enrichment. Research on perceptual load and visual clutter shows that complex, dense, or poorly organized visual information can increase attentional demands and impair visual search (Lavie, 2005; Rosenholtz et al., 2007). Environmental complexity may support preference and orientation only within an appropriate range; excessive or ambiguous complexity can undermine cognitive friendliness (Cassarino and Setti, 2016). Moreover, restorative responses to nature should not be treated as automatic or universal, because the theoretical assumptions behind human preference for green or natural environments have also been critically discussed in previous restorative-environment research (Joye and van den Berg, 2011). Natural environments are not always restorative when their spatial qualities create uncertainty or reduce perceived safety (Gatersleben and Andrews, 2013). For this reason, the present intervention deliberately used restrained, low-saturation, nature-inspired imagery rather than highly saturated decorative content, complex advertising, or dense multimedia displays.

Attention Restoration Theory provides a more specific explanation for this design choice. The intervention was designed to approximate the four restorative dimensions under the spatial constraints of metro stations. First, soft fascination was provided by calm natural imagery that could attract attention without demanding effortful processing. Second, being away was approximated by brief visual cues of nature within an otherwise routine underground commute. Third, extent was supported by coherent imagery that visually connected display surfaces or wall panels rather than presenting fragmented objects. Fourth, compatibility was addressed by using low-interference imagery that remained compatible with commuting tasks, wayfinding, safety information, and movement through the station. These dimensions justify why the study tested minimalist digital natural visuals rather than visually intense environmental decoration.

*H1*: Commuters exposed to the nature-inspired visual intervention condition will report higher momentary affective well-being than those exposed to the conventional visual environment condition.

Perceived crowding refers to the subjective feeling that a space is restrictive, congested, or psychologically pressured by the presence of other people. Density is an objective spatial condition, whereas crowding is a psychological experience (Stokols, 1972). This distinction is important in metro environments because passengers may experience the same number of people differently under different environmental conditions. Rail passenger crowding is associated with stress and exhaustion, and it should be measured as a psychological experience rather than only as physical density (Mohd Mahudin et al., 2012). Evans and Wener (2007) also found that personal space invasion in train environments was related to commuter stress. Wang and Zacharias (2020) further reported that perceived crowding in public transport is influenced by passenger density together with environmental perceptions such as noise and odor.

A nature-inspired visual intervention may also influence perceived crowding in metro stations. A visually monotonous station may make passenger presence and spatial restriction more salient. In contrast, a visually coherent station with calm restorative cues may make the same passenger density feel more acceptable. Public transport design research has shown that supportive design features can reduce unpleasantness and buffer subjective crowding experiences under crowded conditions (Lombardi and Ciceri, 2021). Because the present experiment held passenger number and position constant across paired images, it allowed the study to examine whether a change in restorative visual content and color tone could alter subjective crowding appraisals. Therefore, the intervention may reduce commuters’ perceived crowding, which may further improve their affective well-being.

*H2*: Perceived crowding mediates the relationship between experimental visual condition and commuters’ momentary affective well-being.

Environmental comfort refers to commuters’ subjective feelings of ease, relaxation, pleasantness, and suitability when experiencing a metro station environment. In the present study, environmental comfort is limited to psychologically perceived comfort based on visual experience. It does not refer to objectively measured thermal comfort, acoustic comfort, or air quality. This definition is appropriate because the study uses controlled visual stimuli. Comfort is an important part of passenger experience in metro environments. EEG-based field research has shown that passengers respond differently to comfortable and uncomfortable subway station environments (Kim et al., 2020). Public transport research has also shown that environmental conditions and design resources influence travel comfort and pleasantness (Lombardi and Ciceri, 2021; Wang and Zacharias, 2020). The tested visual intervention may improve environmental comfort in several ways. Harmonious low-saturation colors may reduce visual irritation, and previous research has shown that light and color conditions in indoor environments can influence psychological mood and affective responses (Küller et al., 2006). Nature-inspired digital displays may provide a mild restorative experience. Coherent visual content may make waiting and moving through a station feel easier, even when the actual layout is unchanged. These effects are consistent with studies showing that natural visual exposure supports stress recovery and psychological restoration (Hartig et al., 2003; Kaplan, 1995; Ulrich et al., 1991). Therefore, commuters may feel more comfortable in station scenes containing restrained restorative visual cues, and this comfort may improve their momentary affective well-being.

*H3*: Environmental comfort mediates the relationship between experimental visual condition and commuters’ momentary affective well-being.

Perceived crowding and environmental comfort may also function as a sequential psychological pathway. Conceptually, perceived crowding is treated as a spatial-pressure appraisal that is closely linked to the salience of other passengers and personal space. Environmental comfort is treated as a broader evaluative response that can integrate this spatial-pressure appraisal with the pleasantness and usability of the visual environment. Passengers who experience a metro station as visually monotonous may feel stronger spatial pressure from other people; this perceived crowding may then reduce their evaluation of environmental comfort, and lower comfort may finally lead to poorer momentary affective well-being. In contrast, a restrained nature-inspired visual intervention may reduce perceived crowding, improve environmental comfort, and support more positive emotional states. This pathway is consistent with the stimulus–organism–response framework, in which environmental stimuli influence emotional responses through internal psychological evaluations (Mehrabian and Russell, 1974), and with public transport research showing that crowding, comfort, pleasantness, and travel well-being are closely related (Ettema et al., 2012; Friman et al., 2017; Lombardi and Ciceri, 2021). Because both mediators are measured after exposure to the visual stimuli, the proposed sequence should be interpreted as a theoretically specified appraisal pathway rather than definitive evidence of temporal causality.

*H4*: Perceived crowding and environmental comfort sequentially mediate the relationship between experimental visual condition and commuters’ momentary affective well-being.

To test these hypotheses, this study developed an image-based between-subjects experiment. Three common metro station settings were selected: a platform waiting area, a transfer corridor, and a concourse and fare-gate transition area. Each setting included a conventional visual environment condition and a nature-inspired visual intervention condition. The intervention condition used coherent nature-inspired digital visual content and low-saturation green or blue-green tones. Spatial layout, passenger number, passenger position, lighting, signage, viewing perspective, and facility arrangement were kept constant between paired images. This design allowed the study to examine the influence of a targeted restorative visual intervention while controlling other major environmental differences.

**Figure 1:**
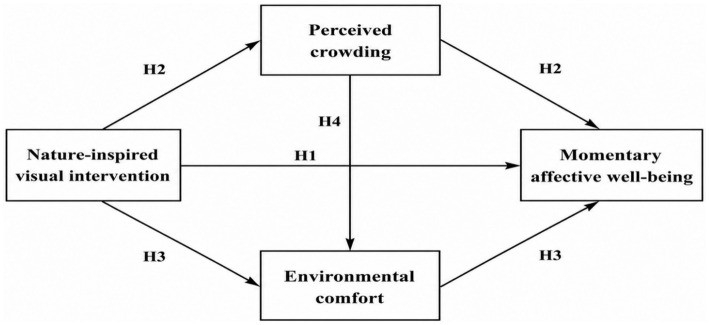
The proposed serial mediation model.

By doing so, this study extends metro station research from operational efficiency and passenger satisfaction to commuters’ immediate affective well-being. It also clarifies the psychological roles of perceived crowding and environmental comfort in linking a targeted nature-inspired visual intervention with affective experience. In addition, the study tests a sequential mediation pathway in which the intervention may reduce perceived crowding, increase environmental comfort, and enhance momentary affective well-being. The proposed research model is shown in Figure 1.

## 2 Materials and methods

### 2.1 Research design and stimuli

**Figure 2:**
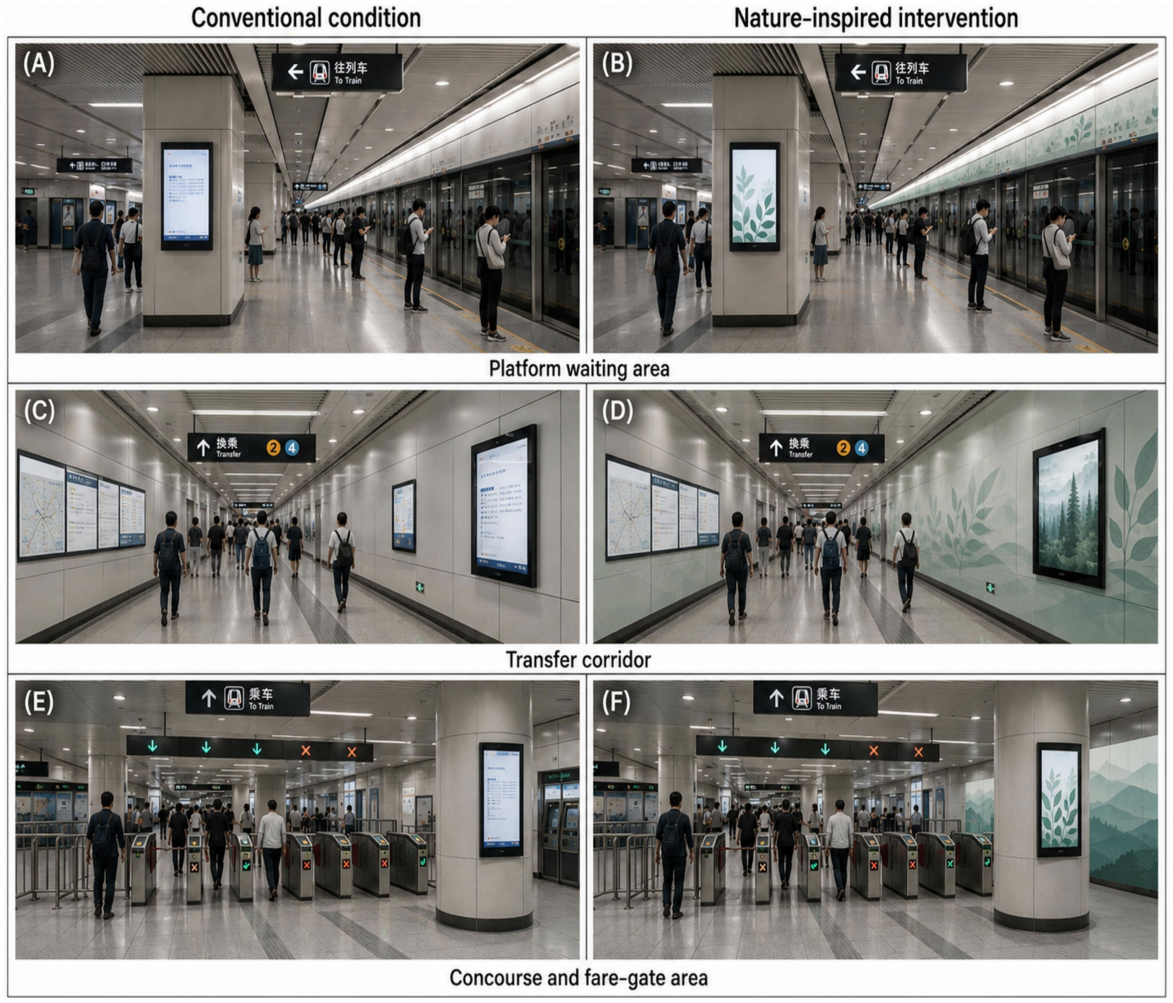
**A-F** Experimental visual stimuli representing the conventional visual environment and nature-inspired visual intervention conditions across three metro station settings.

This study employed a one-factor, between-subjects experimental design to examine the effect of a restrained nature-inspired visual intervention on commuters’ momentary affective well-being. Based on the stimulus–organism–response framework, experimental visual condition served as the environmental stimulus, perceived crowding and environmental comfort as psychological evaluations, and momentary affective well-being as the affective outcome. Participants were randomly assigned to either a conventional visual environment condition (control) or a nature-inspired visual intervention condition, and each participant viewed images from only one condition. The stimuli comprised six images depicting three common metro station spaces: a platform waiting area, a transfer corridor, and a concourse and fare-gate transition area. For each space, the conventional version presented a clean and functional station environment with neutral surfaces and ordinary display content, whereas the intervention version presented the same setting with coherent nature-inspired digital imagery and harmonious low-saturation green or blue-green tones. The six experimental stimuli are presented in Figure 2.

To ensure experimental control, each intervention image was developed as a visual modification of its corresponding conventional image. Spatial layout, viewing perspective, lighting, passenger number and position, facilities, signage, display locations, and basic station configuration remained constant across each image pair; only the visual content and color coordination of the designated display or wall-surface areas differed. Thus, the experiment did not independently manipulate other possible visual-environment dimensions such as material quality, lighting quality, signage design, visual hierarchy, or information organization. All images were presented in a horizontal 16:9 format from an adult eye-level perspective and depicted an ordinary weekday commuting situation with a moderate level of passenger presence (see Table 1).

**Table 1:** Experimental visual conditions.

Original item	Conventional condition	Nature-inspired intervention	Controlled elements and parameters
Platform waiting area	Neutral surfaces; ordinary display content	Nature imagery; green/blue-green tones	Controlled: layout, passengers, lighting, signage, viewpoint. Parameters: Hue-Saturation-Value (HSV) S = 20–32%; nature coverage = 8–12%; no dense text or high-contrast advertising.
Transfer corridor	Neutral wall displays	Continuous restorative visual content	Controlled: corridor form, passengers, lighting, signage, viewpoint. Parameters: HSV S = 18–30%; nature coverage = 14–18%; repeated organic imagery; no added visual clutter.
Concourse and fare-gate area	Neutral visual displays	Restorative imagery in display areas	Controlled: gates, pillars, passengers, lighting, signage, viewpoint. Parameters: HSV S = 20–34%; nature coverage = 8–15%; signage hierarchy unchanged.

The visual parameters in this table are stimulus-development records intended to improve replicability. Hue-Saturation-Value (HSV) saturation values refer to approximate mean saturation in the altered display or wall-surface regions; coverage ratios refer to the proportion of visible image area occupied by the added natural visual content.

### 2.2 Ecological validity of static 2D stimuli

Static 2D pictures were used to isolate the visual intervention as the single manipulated stimulus. This design strengthened internal validity by holding layout, passenger number, lighting, signage, facilities, and viewing perspective constant across paired images. The use of photographs, photorealistic renderings, and other static image-based stimuli has a long methodological basis in environmental psychology, landscape assessment, and urban-design research. Previous studies have used such stimuli to examine environmental preference, visual perception, perceived usability, and related psychological judgments. Meta-analytic and validation evidence suggests that photograph-based evaluations can correspond closely with in-situ environmental appraisals under appropriate conditions, and recent urban visual perception studies further show that image-based surveys can support controlled and reproducible assessment of perceived environmental qualities (Stamps, 1990; Coeterier, 1983; Acemyan and Kortum, 2018; Gu et al., 2025).

However, image-based methods also involve an acknowledged trade-off between experimental control and ecological validity. Validation studies comparing photographs, panoramic images, virtual reality, and field exposure indicate that off-site visual approaches are not always equivalent to direct environmental experience and that their reliability may vary according to presentation medium, environmental type, season, image quality, viewing perspective, and the degree of sensory immersion (Higuera-Trujillo et al., 2017; Xiang et al., 2021). Actual metro experience is multisensory and dynamic: passengers are exposed to train noise, announcements, temperature, air quality, tactile movement, varying crowd flows, time pressure, and changing platform events. Therefore, the present design should be understood as a controlled test of visual appraisal rather than a full simulation of metro travel. The trade-off was intentional: static images made it possible to examine whether a carefully bounded visual change could alter perceived crowding, comfort, and affective appraisal before moving to virtual-reality or field experiments.

Overall, the static-image design was used to maximize internal validity and isolate the visual intervention, whereas future virtual-reality and field studies are needed to test whether the same psychological effects emerge under dynamic and multisensory commuting conditions.

For future virtual-reality and field experiments, standardized data-retention protocols should be implemented to strengthen replicability and allow more complete robustness testing. At minimum, follow-up studies should retain anonymized participant IDs, randomization-order logs, exposure duration for each scene, device or screen parameters, time stamps, viewing-distance or headset records, and contextual environmental indicators such as lighting, noise, temperature, passenger-flow level, and field-station location. These records would make it possible to test order effects, dose–response relationships, and the stability of the visual-intervention effect across realistic transit conditions.

### 2.3 Participants and procedure

Participants were recruited from adults who had used the metro for commuting at least once per week during the previous 6 months in Ningbo, China. To be eligible for the study, participants were required to be at least 18 years old, have used the metro at least once per week during the previous 6 months, have normal or corrected-to-normal vision, and be able to complete the questionnaire independently. The study aimed to obtain approximately 360 valid responses, with a broadly balanced allocation across the two experimental conditions. An *a priori* Monte Carlo power analysis indicated that a sample of approximately 360 participants would provide sufficient power (0.80) to detect the proposed indirect effects at a two-tailed significance level of 0.05 (Schoemann et al., 2017).

The online experiment was administered through Credamo, a professional online research platform widely used for questionnaire-based and behavioral studies in China. The platform was used to present the visual stimuli, randomly assign participants to the conventional visual environment or nature-inspired visual intervention condition, implement the randomized presentation of the metro station scenes, and conduct response-quality screening procedures. Eligible participants were then randomly assigned to one of the two conditions. Before image exposure, they received the following instruction:

Before viewing the images, participants were asked to imagine that they were taking an ordinary weekday metro commute. They were instructed to observe each station scene carefully and to report how they would feel while waiting in or moving through the displayed spaces.

**Figure 3:**
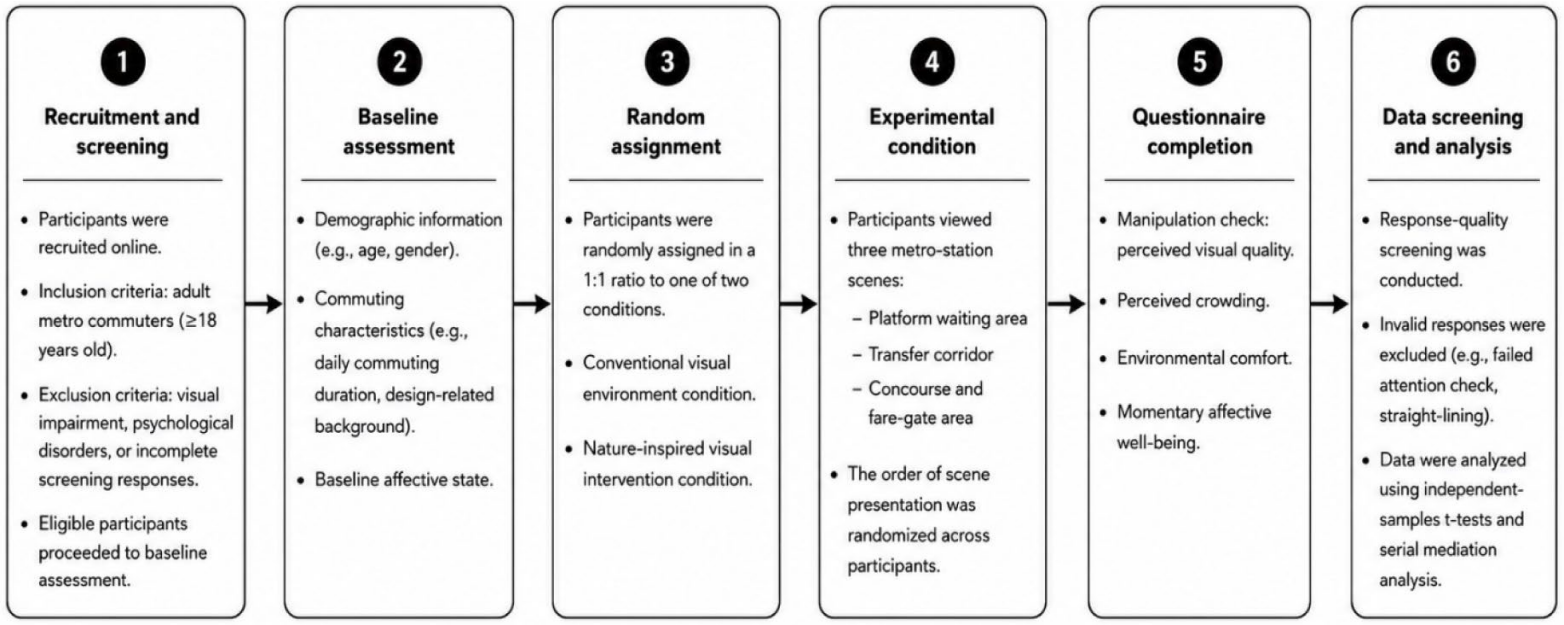
Experimental procedure.

Participants viewed the three images corresponding to their assigned condition. The order of the platform, corridor, and concourse scenes was randomized, and each image remained visible for at least 10 s before participants could proceed. After viewing all three scenes, participants completed the measures of perceived visual quality, perceived crowding, environmental comfort, and momentary affective well-being. An attention-check item and an image-visibility confirmation item were included at the end of the questionnaire. Responses were excluded if participants failed the attention check, did not meet the eligibility criteria, reported that the images were not displayed clearly, provided incomplete responses for the main variables, or completed the questionnaire in an implausibly short time (see Figure 3).

### 2.4 Measures

Unless otherwise stated, items were rated on a 7-point scale. For agreement-based items, responses ranged from 1 (strongly disagree) to 7 (strongly agree). For bipolar affective items, higher scores indicated more positive affective experience. Items adapted from English-language sources were translated into Chinese and then back-translated into English by two bilingual researchers. Discrepancies were discussed and resolved before data collection to ensure semantic equivalence and contextual suitability for Chinese metro commuters. The wording was also checked for clarity, cultural appropriateness, and relevance to metro-station scenes before formal administration.

During instrument development, the adapted Chinese items underwent a procedural pre-test workflow rather than an independently retained psychometric pilot. Specifically, the authors first reviewed each translated and back-translated item for conceptual equivalence, then checked whether the wording could be understood by ordinary metro commuters without specialized design knowledge, and finally examined whether the online questionnaire flow, image display, minimum exposure time, attention-check item, and response format operated normally on the survey platform. Minor wording adjustments were made when items were judged to be ambiguous or insufficiently tailored to the metro-station context.

However, the preliminary checking procedure was used only for item clarity and platform-function verification, and the original item-level pre-test responses were not retained as an independent analyzable dataset. Therefore, preliminary Cronbach’s alpha values, exploratory factor loadings, or pre-test confirmatory factor statistics cannot be reported without reconstructing data that are no longer available. To avoid overstating the evidence, the manuscript reports reliability, convergent validity, and confirmatory factor analysis results from the full formal sample in Section 3.3. This decision improves transparency but also limits the independent documentation of scale adaptation; the issue is therefore acknowledged again in the limitations section, together with a recommendation that future studies retain complete pilot datasets and report pre-test item distributions, factor loadings, reliability coefficients, and item-revision decisions.

Perceived visual quality was measured as a manipulation check rather than as the primary explanatory variable in the mediation model, because the independent variable was the experimentally assigned visual condition. The four visual-quality items assessed whether the station environments appeared aesthetically pleasing, visually harmonious, well organized, and of high visual quality. These items were adapted from research on perceived environmental quality, urban visual evaluation, and restorative urban environments (Nasar, 1994; Ewing and Handy, 2009; Ríos-Rodríguez et al., 2021; Miola and Pazzaglia, 2025).

Perceived crowding was measured using four items adapted from research on rail passenger crowding and public transport design (Mohd Mahudin et al., 2012; Lombardi and Ciceri, 2021). The items captured subjective crowding rather than objective passenger density, including perceived spatial restriction, insufficient personal space, pressure caused by the presence of other passengers, and the overall feeling that the station space was crowded. This construct was treated as the first mediator because it reflects a spatial-pressure appraisal that can emerge immediately after exposure to a station scene.

Environmental comfort was assessed using four context-specific items addressing how comfortable, pleasant, easy to move through or wait in, and suitable for routine commuting the station environments appeared. In this study, environmental comfort refers to visually induced psychological comfort rather than thermal, acoustic, or air-quality comfort. It was treated as the second mediator because it represents a broader evaluative appraisal that integrates perceived spatial pressure with the pleasantness and usability of the visual environment.

Momentary affective well-being was assessed using affective adjective pairs adapted to the metro commuting context from the affective component of the Satisfaction with Travel Scale (Ettema et al., 2011; Friman et al., 2013). Participants were asked how they would feel if they were commuting through the displayed station environments at that moment. Five bipolar items were used: stressed-calm, tense-relaxed, tired-refreshed, bored-engaged, and unpleasant-pleasant. Higher mean scores indicated better momentary affective well-being. The construct was selected because the experiment focused on short-term emotional responses to visual station scenes rather than long-term life satisfaction or clinical mental health.

Baseline affective state was measured before image exposure using three brief self-developed items concerning current relaxation, stress, and mental fatigue. These items were used as a control variable because pre-existing relaxation, stress, and fatigue may influence how participants evaluate the subsequent visual stimuli. The three items were not intended to form a full clinical or trait affect scale. Rather, they were designed to capture immediate pre-exposure affective conditions that could be statistically controlled. The full wording, response format, and reverse-scoring rules of the baseline affective-state items are provided in Supplementary Table S1 (see Table 2).

**Table 2:** Measures used in the study.

Construct	Role	Proposed items	Response format	Main source basis
Perceived visual quality	Manipulation check	Aesthetic; harmonious; organized; high visual quality	7-point agreement scale	Ríos-Rodríguez et al. (2021), Miola and Pazzaglia (2025)
Perceived crowding	Mediator 1	Crowded; spatially restricted; insufficient personal space; pressured by other passengers	7-point agreement scale	Mohd Mahudin et al. (2012), Lombardi and Ciceri (2021)
Environmental comfort	Mediator 2	Comfortable; pleasant; easy to move through or wait in; suitable for routine commuting	7-point agreement scale	Wang and Zacharias (2020), Ríos-Rodríguez et al. (2021)
Momentary affective well-being	Dependent variable	Stressed–calm; tense–relaxed; tired–refreshed; bored–engaged; unpleasant–pleasant	7-point bipolar scale	Ettema et al. (2011), Friman et al. (2013)
Baseline affective state	Control variable *	Relaxed; stressed; mentally fatigued	7-point agreement scale	Developed for the present study

The complete wording, response format, and scoring rules of the baseline affective-state items are reported in Supplementary Table S1.

### 2.5 Data analysis

Data analysis was conducted in four steps. First, SPSS was used for data screening, descriptive statistics, manipulation checks, reliability analysis, and independent-samples *t*-tests. Second, Amos was used to conduct confirmatory factor analysis and assess the measurement model. Third, the direct effect of experimental condition on momentary affective well-being was examined. Finally, PROCESS Model 6 was used to test the separate and sequential mediating effects of perceived crowding and environmental comfort.

The measurement properties of the multi-item constructs were examined. Internal consistency was assessed using Cronbach’s alpha and composite reliability, with values of 0.70 or above regarded as acceptable. Confirmatory factor analysis was used to evaluate whether perceived crowding, environmental comfort, and momentary affective well-being were empirically distinguishable. Model fit was assessed using the comparative fit index (CFI), Tucker–Lewis index (TLI), root mean square error of approximation (RMSEA), and standardized root mean square residual (SRMR). Convergent validity was evaluated using standardized factor loadings and average variance extracted (AVE).

Effect sizes were interpreted in addition to *p*-values to distinguish statistical significance from practical design relevance. Cohen’s d values from group comparisons were considered alongside the raw mean differences on the 7-point scales, following recommendations for reporting and interpreting effect sizes in psychological research (Lakens, 2013). Because the mediators and outcome were measured in the same post-exposure questionnaire, the serial mediation model was specified as a theoretically ordered appraisal pathway rather than as a longitudinal causal process. This interpretation is consistent with methodological cautions regarding cross-sectional mediation analysis (Maxwell and Cole, 2007).

The hypothesized relationships were tested using serial mediation analysis. Experimental condition was entered as the independent variable, coded as 0 for the conventional visual environment condition and 1 for the nature-inspired visual intervention condition. Perceived crowding was entered as the first mediator, environmental comfort as the second mediator, and momentary affective well-being as the dependent variable. Baseline affective state, age, gender, daily commuting duration, and design-related background were included as covariates. The analysis estimated the direct effect of experimental condition on momentary affective well-being, the separate indirect effects through perceived crowding and environmental comfort, and the sequential indirect effect through perceived crowding followed by environmental comfort. Indirect effects were tested using 5,000 bootstrap resamples and bias-corrected 95% confidence intervals. An indirect effect was regarded as significant when the confidence interval did not include zero.

Additional robustness analyses were conducted to report covariate predictive effects and exploratory subgroup differences by design-related background. Covariate paths were examined for perceived crowding, environmental comfort, and momentary affective well-being. The serial mediation model was also estimated separately for participants with and without a design-related background. The presentation order of the three metro-station scenes was randomized during stimulus exposure to reduce systematic order bias. However, because the anonymized analysis dataset did not retain individual scene-viewing sequence identifiers, a *post hoc* statistical test of scene-order effects could not be conducted from the available dataset.

## 3 Results

### 3.1 Participant characteristics

A total of 380 responses were collected in the experimental survey. After excluding responses that failed the attention check, did not meet the eligibility criteria, contained incomplete answers for the primary variables, or showed abnormal completion patterns, 360 valid responses were retained for analysis. Of these participants, 180 were assigned to the conventional visual environment condition and 180 were assigned to the nature-inspired visual intervention condition.

The mean age of the valid sample was 29.40 years (SD = 7.21). The sample included 180 male participants (50.0%), 174 female participants (48.3%), and 6 participants who selected another option or preferred not to report their gender (1.7%). The mean daily commuting duration was 45.10 min (SD = 15.65). In addition, 50 participants (13.9%) reported a professional or educational background related to design or similar fields. The demographic and commuting characteristics of the participants are summarized in Table 3.

**Table 3:** Participant characteristics.

Characteristic	Total sample	Conventional	Intervention
Number of valid participants, *n*	360	180	180
Age, M (SD)	29.40 (7.21)	28.76 (6.86)	30.04 (7.51)
Male, *n* (%)	180 (50.00%)	89 (49.44%)	91 (50.56%)
Female, n (%)	174 (48.33%)	90 (50.00%)	84 (46.67%)
Other / prefer not to say, *n* (%)	6 (1.67%)	1 (0.56%)	5 (2.78%)
Daily commuting duration, M (SD), min	45.10 (15.65)	44.46 (15.45)	45.74 (15.86)
Design-related background, *n* (%)	50 (13.89%)	31 (17.22%)	19 (10.56%)

### 3.2 Manipulation check

An independent-samples *t*-test was conducted to determine whether the two experimental conditions differed in perceived visual quality. Participants in the nature-inspired visual intervention condition reported substantially higher perceived visual quality (M = 4.89, SD = 0.71) than those in the conventional visual environment condition (M = 3.68, SD = 0.69), t(358) = 16.38, *p* < 0.001, Cohen’s d = 1.73. This large difference confirmed that the intervention increased perceived visual quality. Importantly, the conventional condition represented a clean, functional, and relatively standard modern metro station environment rather than a deliberately poor or low-quality setting (see Table 4).

**Table 4:** Manipulation check for perceived visual quality.

Variable	Conventional M (SD)	Intervention M (SD)	*t*	*p*	Cohen’s d
Perceived visual quality	3.68 (0.69)	4.89 (0.71)	16.38	<0.001	1.73

**Figure 4:**
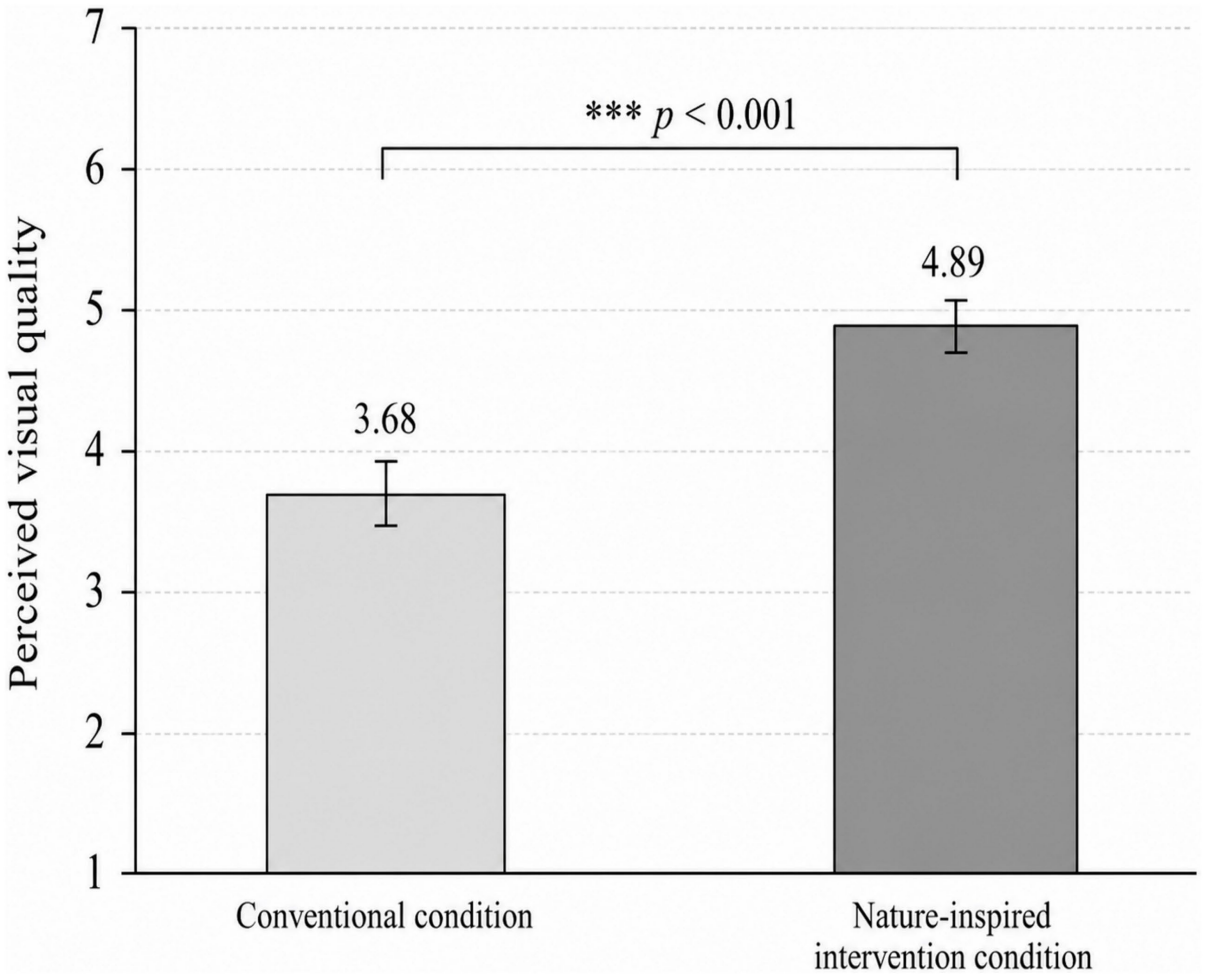
Manipulation check results for perceived visual quality across the two experimental conditions.

As shown in Figure 4, participants clearly distinguished the nature-inspired intervention scenes from the conventional scenes in terms of perceived visual quality. This result supports the use of the two conditions in the subsequent hypothesis testing while avoiding the misleading implication that the control condition was intrinsically “low quality.”

### 3.3 Reliability and validity

The reliability and validity of the three primary psychological constructs were assessed before testing the hypothesized relationships. As shown in Table 5, Cronbach’s alpha values ranged from 0.86 to 0.90, and composite reliability values ranged from 0.86 to 0.90, exceeding the recommended threshold of 0.70. The standardized factor loadings ranged from 0.75 to 0.83, and the average variance extracted (AVE) values ranged from 0.61 to 0.65, indicating satisfactory convergent validity.

**Table 5:** Reliability and convergent validity of the study constructs.

Construct	Item	Standardized loading	Cronbach’s α	CR	AVE
Perceived crowding	PC1	0.83	0.88	0.88	0.64
	PC2	0.77			
	PC3	0.82			
	PC4	0.79			
Environmental comfort	EC1	0.77	0.86	0.86	0.61
	EC2	0.75			
	EC3	0.80			
	EC4	0.80			
Momentary affective well-being	AWB1	0.81	0.90	0.90	0.65
	AWB2	0.82			
	AWB3	0.80			
	AWB4	0.81			
	AWB5	0.78			

α, Cronbach’s alpha; CR, composite reliability; AVE, average variance extracted; CFI, comparative fit index; TLI, Tucker-Lewis index; RMSEA, root mean square error of approximation; SRMR, standardized root mean square residual.

A three-factor confirmatory factor analysis was conducted for perceived crowding, environmental comfort, and momentary affective well-being. The proposed measurement model demonstrated an acceptable fit to the data, χ^2^/df = 0.98, CFI = 1.00, TLI = 1.00, RMSEA = 0.000, and SRMR = 0.027. These results indicate that the three constructs were empirically distinguishable and suitable for subsequent direct-effect and mediation analyses.

### 3.4 Group differences and direct effect

**Figure 5:**
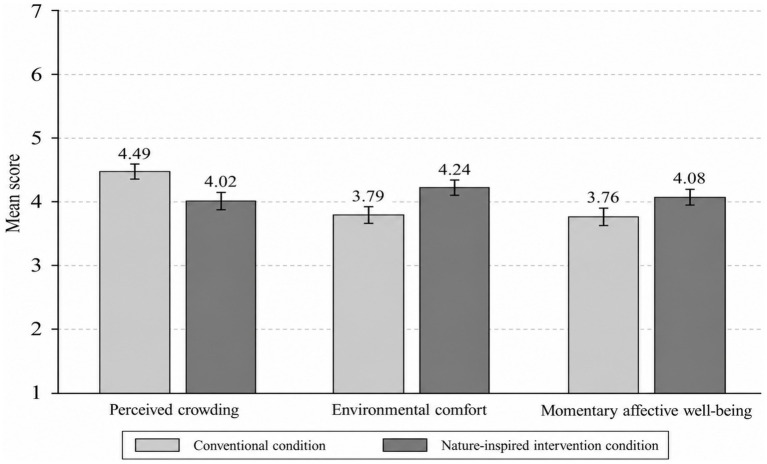
Mean differences in perceived crowding, environmental comfort, and momentary affective well-being between the two experimental conditions. Error bars represent standard errors.

Table 6 reports the group differences between the two experimental conditions. Relative to the conventional visual environment condition, the nature-inspired visual intervention condition was associated with lower perceived crowding, t(358) = −6.00, *p* < 0.001, Cohen’s d = −0.63, higher environmental comfort, t(358) = 5.92, *p* < 0.001, Cohen’s d = 0.62, and higher momentary affective well-being, t(358) = 3.73, *p* < 0.001, Cohen’s d = 0.39. These findings supported Hypothesis 1 and provided preliminary support for the proposed mediation analysis (see Figure 5).

**Table 6:** Mean differences in perceived crowding, environmental comfort, and momentary affective well-being between the two experimental conditions.

Outcome	Conventional M (SD)	Intervention M (SD)	*t*	*p*	Cohen’s d
Perceived crowding	4.49 (0.73)	4.02 (0.74)	−6.00	< 0.001	−0.63
Environmental comfort	3.79 (0.75)	4.24 (0.71)	5.92	< 0.001	0.62
Momentary affective well-being	3.76 (0.85)	4.08 (0.78)	3.73	< 0.001	0.39

From a practical design perspective, these effect sizes indicate moderate reductions in perceived crowding and moderate increases in environmental comfort, together with a small-to-moderate improvement in momentary affective well-being. On the 7-point scales, the intervention reduced perceived crowding by 0.47 points and increased environmental comfort by 0.45 points, while affective well-being increased by 0.32 points. These changes should not be interpreted as substitutes for capacity expansion, passenger-flow management, or operational improvements. However, because the intervention relied on existing display and wall-surface areas rather than structural reconstruction, the results suggest meaningful low-cost potential for improving passengers’ immediate visual and emotional experience.

### 3.5 Mediation and serial mediation effects

A serial mediation analysis was conducted using PROCESS Model 6 with 5,000 bootstrap resamples. Experimental condition was coded as 0 for the conventional visual environment condition and 1 for the nature-inspired visual intervention condition. Perceived crowding was entered as the first mediator, environmental comfort as the second mediator, and momentary affective well-being as the dependent variable. Baseline affective state, age, gender, daily commuting duration, and design-related background were included as covariates.

**Figure 6:**
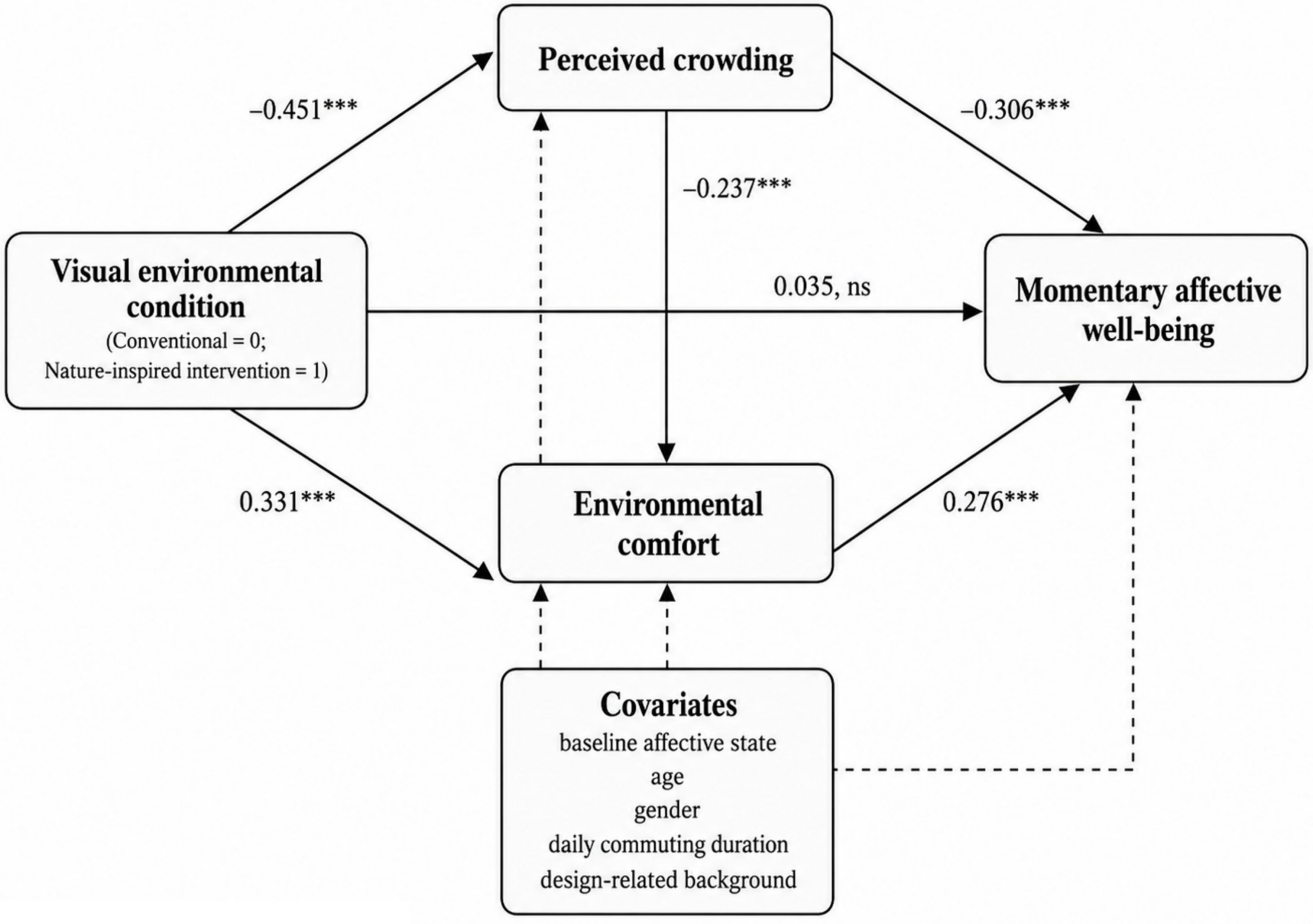
Estimated path coefficients for the serial mediation model. Experimental condition was coded as 0 = conventional visual environment and 1 = nature-inspired visual intervention. Solid arrows indicate the primary mediation paths, and dashed arrows indicate covariate paths. *** *p* < 0.001; ns, non-significant.

Figure 6 presents the estimated path coefficients for the serial mediation model. The experimental condition significantly predicted perceived crowding, *B* = −0.451, SE = 0.078, *p* < 0.001, indicating that participants in the nature-inspired visual intervention condition reported lower perceived crowding than those in the conventional condition. The experimental condition also significantly predicted environmental comfort, *B* = 0.331, SE = 0.078, *p* < 0.001, indicating that the intervention increased perceived comfort. In addition, perceived crowding negatively predicted environmental comfort, *B* = −0.237, SE = 0.051, *p* < 0.001, suggesting that stronger perceived crowding was associated with lower environmental comfort.

The two mediators also showed significant associations with momentary affective well-being. Perceived crowding negatively predicted momentary affective well-being, *B* = −0.306, SE = 0.053, t = −5.78, *p* < 0.001, whereas environmental comfort positively predicted momentary affective well-being, *B* = 0.276, SE = 0.054, t = 5.14, *p* < 0.001. These path coefficients indicate that commuters’ affective responses were related not only to the assigned visual condition but also to how crowded and comfortable the station environment was perceived to be.

After perceived crowding and environmental comfort were included in the model, the direct effect of experimental condition on momentary affective well-being was no longer significant, *B* = 0.035, SE = 0.080, t = 0.43, *p* = 0.668. This result indicates that the effect of the nature-inspired visual intervention on affective well-being was largely accounted for by the proposed psychological appraisal process. In other words, the intervention improved affective well-being mainly by changing participants’ perceptions of crowding and environmental comfort rather than by exerting a remaining direct effect independent of these mediators.

Table 7 reports the bootstrap tests of the indirect effects. The indirect effect through perceived crowding was significant, effect = 0.138, bootstrap standard error (Boot SE) = 0.034, 95% confidence interval (CI) [0.079, 0.212], supporting Hypothesis 2. The indirect effect through environmental comfort was also significant, effect = 0.091, Boot SE = 0.027, 95% CI [0.043, 0.149], supporting Hypothesis 3. The sequential indirect effect through perceived crowding and environmental comfort was significant as well, effect = 0.030, Boot SE = 0.010, 95% CI [0.013, 0.052], supporting Hypothesis 4. Because all confidence intervals excluded zero, the results supported both separate and serial mediation effects. However, the serial pathway should be interpreted as a theoretically specified appraisal process rather than as proof of temporal causality between the two mediators.

**Table 7:** Bootstrap tests of indirect effects.

Indirect pathway	Effect	Boot SE	LLCI	ULCI	Result
Experimental condition → perceived crowding → affective well-being	0.14	0.03	0.08	0.21	Supported
Experimental condition → environmental comfort → affective well-being	0.09	0.03	0.04	0.15	Supported
Experimental condition → perceived crowding → environmental comfort → affective well-being	0.03	0.01	0.01	0.05	Supported
Total indirect effect	0.26	0.04	0.18	0.35	Significant

Boot SE, bootstrap standard error; LLCI, lower limit of the 95% confidence interval; ULCI, upper limit of the 95% confidence interval.

**Figure 7:**
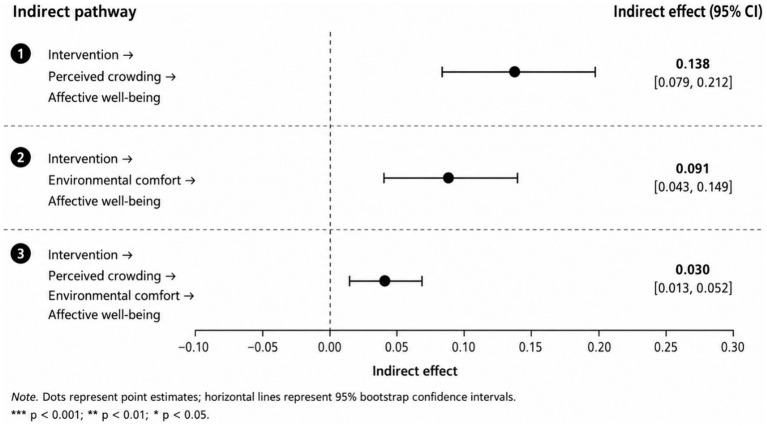
Bootstrap estimates and 95% confidence intervals for the indirect effects on momentary affective well-being.

The pattern shown in Table 7 and Figure 7 indicates that the indirect pathway through perceived crowding had the largest effect among the three specific indirect effects, followed by the pathway through environmental comfort and the sequential pathway. The total indirect effect was also significant, effect = 0.259, Boot SE = 0.044, 95% CI [0.177, 0.351]. Taken together, these findings suggest that the nature-inspired visual intervention may improve commuters’ momentary affective well-being through a connected appraisal process: the intervention reduces perceived crowding, lower perceived crowding contributes to greater environmental comfort, and greater environmental comfort is associated with more positive affective experience.

The practical meaning of the indirect effects should also be interpreted cautiously and in relation to the low-cost nature of the tested visual retrofit. The indirect pathway through perceived crowding was the largest specific effect, suggesting that the main applied value of the intervention may lie in reducing subjective spatial pressure rather than merely improving decorative appearance. The environmental-comfort pathway was smaller but still meaningful because comfort is a directly designable appraisal that can be influenced through wall-surface imagery, screen content, and color coordination. The sequential pathway was the smallest effect, indicating that the chain from lower crowding to higher comfort and then to better affective well-being should be viewed as a modest but theoretically informative process. On a 7-point scale, these indirect magnitudes do not represent large emotional changes; nevertheless, they may be practically relevant because screen and wall-surface visual updates are comparatively inexpensive, reversible, and easier to implement than capacity expansion, station reconstruction, or major passenger-flow redesign. Thus, the observed effects should be understood as small-to-moderate design benefits with favorable cost–benefit potential, not as substitutes for operational or structural improvements.

### 3.6 Additional robustness analyses

Additional robustness analyses were conducted to improve methodological transparency and to examine whether the mediation findings remained stable after considering covariate effects and subgroup differences by design-related background. The randomized scene-viewing procedure was used as a procedural control to reduce systematic order bias during stimulus presentation. However, the anonymized analysis dataset did not retain individual scene-viewing sequence identifiers; therefore, a *post hoc* statistical test of scene-order effects could not be conducted from the available dataset. This limitation is acknowledged in the limitations section, and future experiments should retain randomization-order logs to allow direct order-effect testing.

The covariates included in the serial mediation model were examined for transparency. Baseline affective state, age, gender, daily commuting duration, and design-related background were entered as covariates predicting perceived crowding, environmental comfort, and momentary affective well-being. Baseline affective state significantly predicted perceived crowding, B = −0.135, SE = 0.046, *p* = 0.003, environmental comfort, B = 0.104, SE = 0.045, *p* = 0.021, and momentary affective well-being, B = 0.209, SE = 0.045, *p* < 0.001. By contrast, age, gender, daily commuting duration, and design-related background did not show consistent significant predictive effects across the model. These results indicate that the main mediation findings were not primarily driven by demographic or commuting-related covariates.

An exploratory subgroup mediation analysis was also conducted to compare participants with and without a design-related background. This analysis was included only as a supplementary robustness check and should not be interpreted as a confirmatory moderation test. Among participants without a design-related background, the indirect effects through perceived crowding, environmental comfort, and the sequential pathway were all significant. Specifically, the indirect effect through perceived crowding was 0.136, 95% CI [0.072, 0.215], the indirect effect through environmental comfort was 0.092, 95% CI [0.034, 0.160], and the sequential indirect effect was 0.034, 95% CI [0.014, 0.060]. Among participants with a design-related background, the corresponding confidence intervals were wider and included zero, likely because this subgroup was relatively small. The indirect effect through perceived crowding was 0.233, 95% CI [−0.010, 0.559], the indirect effect through environmental comfort was −0.022, 95% CI [−0.337, 0.222], and the sequential indirect effect was −0.005, 95% CI [−0.116, 0.054]. Bootstrap comparisons of subgroup differences showed that none of the differences in indirect effects were statistically reliable. Therefore, the subgroup results do not provide evidence that the mediation mechanism differs systematically by design-related background. Any interpretation of these subgroup findings should remain exploratory and should not be generalized beyond the present sample.

## 4 Discussion

### 4.1 Summary of the main findings

Using an image-based between-subjects experiment, this study examined whether a restrained nature-inspired visual intervention in metro station spaces influences commuters’ momentary affective well-being and whether this relationship is explained by perceived crowding and environmental comfort. The results supported the proposed hypotheses. Participants exposed to the nature-inspired intervention condition reported lower perceived crowding, higher environmental comfort, and higher momentary affective well-being than those exposed to the conventional visual environment condition. The mediation analysis further showed that perceived crowding and environmental comfort explained the relationship between experimental visual condition and affective well-being through both separate and sequential indirect pathways.

These findings indicate that the visual environment of metro stations is not merely a decorative background for commuting. Rather, it forms part of the psychological context through which commuters interpret passenger presence, spatial pressure, comfort, and their own affective state. Because the paired experimental scenes kept spatial layout, passenger number, passenger position, lighting, signage, viewing perspective, and facility arrangement constant, the observed differences are more reasonably interpreted as responses to the tested nature-inspired visual content and color-tone intervention rather than to changes in objective density, station configuration, lighting, or wayfinding design.

To clarify the discussion-level meaning of the results within the boundaries of the tested intervention, Table 8 summarizes the key empirical findings, their psychological interpretation, and their cautious implications for metro station visual design.

**Table 8:** Interpretation of key findings and cautious design implications.

Key finding	Interpretation	Cautious design implication
The intervention improved momentary affective well-being.	Restrained nature-inspired visuals were associated with more positive immediate affective experience.	Such interventions may be used as a complementary passenger-experience strategy, not as evidence for all possible visual design improvements.
The intervention reduced perceived crowding.	Visual context may influence how passengers interpret the same level of crowd presence.	Restorative visual cues may help reduce subjective spatial pressure in dense station areas.
The intervention increased environmental comfort.	Comfort here refers to visually induced psychological comfort, not thermal, acoustic, or air-quality comfort.	Display and wall-surface areas may be used cautiously while maintaining safety and wayfinding clarity.
The serial mediation pathway was significant.	The intervention may influence affective well-being through perceived crowding and comfort appraisals.	Future station evaluations may include crowding perception, visual comfort, and affective experience.

### 4.2 Psychological interpretation of the mediation mechanism

The supported mediation model provides a process-level explanation of why the tested nature-inspired visual intervention matters in metro station settings. The intervention was associated with lower perceived crowding, suggesting that the same number of passengers may be experienced differently depending on the visual context of the station. A visually monotonous station may make passenger presence more salient and spatial pressure more difficult to tolerate. By contrast, a station with restrained restorative cues and low-saturation green or blue-green tones may make shared space feel more manageable.

This interpretation is consistent with the distinction between objective density and perceived crowding. Density refers to the physical number of people within a given space, whereas crowding is a psychological experience shaped by how individuals interpret that space (Stokols, 1972). In public transport settings, crowding has been associated with stress, exhaustion, personal space invasion, and environmental discomfort (Evans and Wener, 2007; Mohd Mahudin et al., 2012; Wang and Zacharias, 2020). The present findings add a visual-environment perspective to this discussion. Even when the passenger environment is held constant, a restrained restorative visual intervention may influence how crowded the same space feels.

Environmental comfort also played an important role in the model. The nature-inspired visual intervention increased environmental comfort, and higher comfort was associated with better momentary affective well-being. This suggests that the intervention may influence emotion not only by reducing negative spatial pressure, but also by increasing positive environmental appraisal. In the metro station context, comfort is not limited to thermal, acoustic, or air-quality conditions. It can also emerge from visually induced perceptions of pleasantness, ease, and suitability for routine commuting.

The sequential pathway further clarifies the internal logic of the proposed model. The intervention reduced perceived crowding, lower perceived crowding contributed to higher environmental comfort, and higher comfort was associated with better affective well-being. This pattern suggests that perceived crowding and environmental comfort are connected appraisals through which commuters evaluate the station environment. Because both mediators were measured at the same post-exposure stage, this sequence should be treated as a theoretically specified psychological pathway rather than definitive evidence that perceived crowding temporally precedes environmental comfort.

### 4.3 Relation to previous research

The findings are consistent with previous studies showing that commuting is an affective experience rather than a purely utilitarian travel activity. Daily travel satisfaction, emotional well-being, and commuting happiness have been shown to be closely related (Bergstad et al., 2011; Ettema et al., 2011; Friman et al., 2017; Olsson et al., 2013). Public transport research has also shown that crowding, limited personal space, waiting, and uncomfortable environmental conditions can reduce travel experience (Evans and Wener, 2007; Gatersleben and Uzzell, 2007; Wang and Zacharias, 2020). The present study extends this literature by showing that a targeted nature-inspired visual intervention in the station environment can influence commuters’ affective evaluation, even when objective passenger conditions are kept constant. This interpretation is also consistent with meta-analytic evidence indicating that perceived crowding can shape emotional and evaluative responses through appraisal-based mechanisms rather than through density alone (Blut and Iyer, 2020).

The results also align with restorative environment research. Natural and visually coherent environments have been shown to support stress recovery and psychological restoration (Hartig et al., 2003; Kaplan, 1995; Ulrich et al., 1991). In this study, the intervention condition did not transform metro stations into natural settings. Instead, it introduced restrained nature-inspired digital imagery and low-saturation green or blue-green tones into existing display areas. The affective benefit observed in the results suggests that restorative visual cues may remain psychologically relevant even in highly functional transport spaces. In addition, systematic and meta-analytic work on nature exposure and attention restoration supports the view that brief contact with natural or nature-like environments can influence affective and cognitive recovery, although the strength of evidence depends on exposure format and outcome type (McMahan and Estes, 2015; Ohly et al., 2016).

The findings also extend recent metro station studies in Chinese and East Asian contexts, but the relationship is not simply additive. EEG-based field research has shown that passengers respond differently to comfortable and uncomfortable subway station environments (Kim et al., 2020), while subway-station greenness research has demonstrated that indoor green visual content can generate psychophysiological responses among users (Kim and Lee, 2021). These studies provide stronger physiological or field-based realism than the present image experiment, but they do not isolate a narrowly bounded nature-inspired screen and wall-surface intervention across controlled passenger, lighting, signage, and layout conditions. By contrast, the present study sacrifices some ecological richness in order to identify a more specific psychological mechanism linking visual condition, perceived crowding, environmental comfort, and affective well-being.

Compared with Chinese metro-design studies, the present contribution is also more mechanism-oriented. Hu et al. (2019) emphasized the optimization of subway station space according to accessibility, convenience, comfort, and cultural experience, whereas the present study operationalizes comfort as a measured psychological mediator and tests its role in an experimental model. Hu and Xu (2023) used a Nanjing metro virtual-reality experiment to examine how guidance signage affects wayfinding performance and environmental pressure; in contrast, the present study does not manipulate wayfinding or signage but focuses on a restrained restorative visual strategy and its affective pathway. Su and Ding (2024) summarized Chinese subway public-space design research and highlighted human-centered, green, cultural, and interdisciplinary directions; the present work translates one of those broad directions into a controlled empirical test. Shi et al. (2025) examined environmental characteristics associated with emotional perception in metro station spaces, while Xie and Li (2026) discussed the integration of Jiangnan cultural elements in metro space. Compared with these broader environmental and cultural-design studies, the present study contributes by distinguishing a specific low-saturation nature-inspired intervention from general visual quality and by showing that its effects are statistically accounted for through perceived crowding and environmental comfort.

Therefore, the divergence between the present findings and prior virtual-reality, field, and design-oriented metro studies lies mainly in the level of causal control and the level of explanatory mechanism. Prior work has been stronger in ecological realism, signage-performance evaluation, psychophysiological measurement, or cultural-design interpretation. The present study is more limited in ecological richness but stronger in isolating a single visual intervention and testing a serial mediation pathway. Its distinctive scholarly contribution is thus not that it offers a comprehensive metro-renewal solution, but that it identifies a bounded restorative visual mechanism that can be examined further in virtual-reality and field experiments.

### 4.4 Theoretical and practical implications

The theoretical value of this study lies in extending the stimulus–organism–response framework to restorative visual interventions in metro station environments. In the proposed model, experimental visual condition functions as the environmental stimulus, perceived crowding and environmental comfort function as organism-level appraisals, and momentary affective well-being functions as the emotional response. The supported indirect paths indicate that visual interventions do not operate as direct aesthetic inputs alone. They influence affective outcomes through commuters’ interpretation of crowding and comfort.

More specifically, the findings support a revised S-O-R framework for dense underground transit environments: restrained visual intervention → spatial-pressure appraisal → comfort appraisal → momentary affective response. In this context, the stimulus is not the entire station environment but a bounded visual strategy embedded within an operationally constrained setting. The organismic process is also not a single general attitude toward the station; it involves layered appraisals, beginning with how passengers interpret the presence of other people and spatial pressure and then extending to comfort and suitability for routine commuting. This transit-specific formulation helps explain why a relatively small visual intervention can matter even when objective density and station layout remain unchanged.

This theoretical interpretation also refines the role of visual design in transport psychology. Rather than treating visual environmental quality as a broad, undifferentiated construct, the present study provides evidence for one specific intervention strategy: restrained nature-inspired imagery combined with low-saturation green or blue-green tones. By reducing perceived crowding and increasing comfort, this type of intervention may influence how commuters experience shared public transport space. This helps connect metro station design research with environmental psychology, crowding research, and travel well-being studies.

The practical implication is that restrained nature-inspired visual interventions can be considered as a complementary strategy for metro station renewal. In many high-density metro systems, increasing station capacity or reducing passenger density requires large-scale investment, engineering reconstruction, operational disruption, and long-term planning. Visual interventions are not a substitute for structural measures, crowd management, safety management, or operational improvements, but they may offer a feasible way to improve perceived comfort in the short term. Existing digital screens, wall display areas, advertising panels, and visual communication surfaces can be used to introduce coherent, low-interference, and restorative visual elements similar to those tested in this study.

The cost–benefit implication of the indirect effects is therefore important. Because the direct effect became non-significant after perceived crowding and environmental comfort were included, practical deployment should not aim simply to make stations appear more decorative. Instead, low-budget visual retrofits should be evaluated according to whether they reduce subjective crowding and improve visually induced comfort. The largest indirect pathway through perceived crowding suggests that even when actual passenger density cannot be reduced immediately, visual context may help passengers appraise the same density as less psychologically pressuring. The smaller comfort and sequential indirect effects indicate that the emotional benefit is likely modest, but the intervention is comparatively low-cost, scalable across repeated commutes, and reversible if it interferes with safety or wayfinding. This balance gives the strategy a potentially favorable applied value when used cautiously alongside operational improvements.

Scenario-specific application is necessary. For peak trunk lines and high-density transfer stations, natural visual interventions should remain low-saturation, low-motion, and low-text, and they should be placed only where they do not interfere with safety information, wayfinding hierarchy, passenger-flow judgment, or emergency communication. In these settings, the design priority should be to reduce subjective spatial pressure without adding visual clutter.

For tourist-oriented or culture-oriented metro stations, nature-inspired visuals may be combined with local landscape imagery, cultural symbols, or city identity elements, but the content should remain visually coherent and should avoid excessive narrative density. Cultural display should not become visual noise. For suburban lines or lower-density stations, where passenger pressure may be lower and waiting time may be longer, future design trials could test more continuous wall-surface imagery, seasonal natural themes, or larger visual-coverage ratios. These differentiated scenarios should be evaluated separately rather than assumed to produce identical psychological effects.

Across all scenarios, visual optimization should remain subordinate to the functional logic of metro stations. Overly saturated colors, dense commercial imagery, visually complex decoration, or excessive digital content may increase cognitive load and weaken comfort. Metro stations are high-intensity public transport spaces where safety, wayfinding clarity, passenger circulation, and accessibility must remain central. For this reason, future station evaluation could include perceived crowding, visually induced environmental comfort, and affective experience alongside traditional indicators such as flow efficiency, safety, wayfinding clarity, and service quality.

### 4.5 Limitations and future research

#### 4.5.1 Ecological and stimulus-related limitations

The study used image-based visual stimuli rather than real metro station exposure. This approach was appropriate for isolating the visual intervention, but it cannot reproduce the dynamic and multisensory conditions of actual commuting, including train noise, announcements, temperature, odor, vibration, time pressure, train arrivals, and changing passenger flows. Because Section 2.2 already provides a detailed methodological justification of static 2D stimuli, this limitation is noted here only to define the boundary of interpretation. Future studies should use virtual reality experiments, field experiments, or before-and-after station renovation studies to examine whether the present effects remain stable in more realistic transit contexts.

#### 4.5.2 Causal limitations of cross-sectional synchronous mediation

Although participants were randomly assigned to the two visual conditions, the mediators and outcome were measured within the same post-exposure questionnaire. Therefore, the serial mediation pathway should be interpreted as a theoretically specified appraisal model rather than as definitive temporal or causal evidence among perceived crowding, environmental comfort, and affective well-being.

First, the design lacks longitudinal temporal sequencing. Perceived crowding, environmental comfort, and momentary affective well-being were not measured at separate time points, so the analysis cannot determine whether perceived crowding actually emerged before comfort appraisal or whether affective state shaped both appraisals simultaneously. A repeated-exposure or experience-sampling design would be needed to test whether changes in crowding appraisal precede later changes in comfort and affect.

Second, the study relied on self-reported psychological responses, which may be affected by common-method bias and social desirability response tendencies. Participants who perceived the intervention scenes as more visually pleasant may have responded more favorably across several measures, and some respondents may have selected answers they considered socially appropriate or consistent with the perceived research purpose. Although the randomized experimental design and attention checks reduced some threats, future work should combine self-report measures with behavioral, physiological, or implicit indicators to reduce reliance on a single response channel (Podsakoff et al., 2003).

Third, stable individual traits may confound the proposed appraisal pathway. Some commuters may be chronically more sensitive to crowding, more tolerant of enclosed underground spaces, more attracted to nature imagery, or more visually design-literate than others. These stable dispositions could influence perceived crowding, comfort, and affective well-being at the same time, even when the visual condition is experimentally controlled. Future research should measure and model crowding sensitivity, trait negative affectivity, environmental preference, nature relatedness, claustrophobic tendency, and visual-design literacy as potential moderators or covariates.

#### 4.5.3 External validity and intervention generalizability

The sample consisted of adult commuters in Ningbo, China, and therefore the results may not directly generalize to larger transfer systems, older metro networks, tourist-oriented stations, suburban rail-transit lines, or metro systems in other cultural and climatic contexts. The tested intervention was also a single combined visual strategy, namely static nature-inspired imagery with low-saturation green or blue-green tones. The study did not independently manipulate warm tones, physical greenery, dynamic displays, natural sound, cultural imagery, screen coverage, lighting, signage, visual hierarchy, or material quality. Therefore, the findings should not be interpreted as evidence for all possible metro-station visual design improvements. Future research should define one comparison dimension at a time, such as image content under the same medium and color tone, color saturation under the same image content, static versus dynamic media under the same visual theme, or physical greenery versus digital imagery.

#### 4.5.4 Measurement, order-effect, and subgroup limitations

The present model relied on self-reported psychological responses and did not include objective physiological or behavioral indicators. Eye-tracking, fixation entropy, skin conductance, heart-rate variability, cortisol or other stress biomarkers, walking-speed data, and dwell-time records would help determine whether the subjective effects observed here are accompanied by objective attentional or stress-related responses. In addition, the anonymized analysis dataset did not retain individual scene-viewing sequence identifiers, which made it impossible to conduct a *post hoc* statistical test of order effects. Future online, virtual-reality, and field experiments should retain randomization-order logs so that possible scene-order effects can be directly examined and reported. Finally, the design-related subgroup was relatively small, and all corresponding subgroup confidence intervals included zero. The subgroup analysis should therefore be treated as exploratory only and should not be generalized to broader populations. Larger and more balanced samples are needed to examine whether design literacy moderates the effects of restorative visual interventions. Because an independent psychometric pre-test dataset was not retained, future studies should also retain complete pilot records after translation and back-translation, including item-level data, reliability estimates, factor loadings, item-revision logs, and platform-function checks.

## 5 Conclusion

This study demonstrates that restrained nature-inspired visual interventions can shape commuters’ momentary affective experience in metro station environments through appraisals of perceived crowding and environmental comfort. The theoretical contribution is to refine the stimulus-organism-response framework for dense underground transit settings by identifying a bounded pathway from a specific visual intervention to spatial-pressure appraisal, comfort appraisal, and affective response. The practical contribution is to show that existing digital screens and wall-surface areas may support low-cost, reversible, and scenario-sensitive passenger-experience improvements when the visual content remains coherent, low-saturation, and subordinate to wayfinding, safety, accessibility, and operational clarity. Future virtual-reality and field studies should test whether this controlled image-based mechanism remains stable in dynamic and multisensory commuting conditions.

## Data Availability

The original contributions presented in the study are included in the article/supplementary material, further inquiries can be directed to the corresponding author.
