# Supplementary material for: Nature-inspired visual interventions in metro stations and commuters’ affective well-being: the mediating roles of perceived crowding and environmental comfort
Source: Front Psychol. 2026 Aug 4;17:1901802. doi: 10.3389/fpsyg.2026.1901802 (PMC13481186; doi:10.3389/fpsyg.2026.1901802)
Supplement: Supplementary file 1 [file Data_Sheet_1.PDF]

# Supplementary Tables S1-S3

## Manuscript ID: 1901802

Nature-Inspired Visual Interventions in Metro Stations and Commuters' Affective Well-Being: The Mediating Roles of Perceived Crowding and Environmental Comfort

*Note.* The supplementary tables have been reformatted according to the reviewer's request. Each table now includes a unified title with manuscript ID, table-specific notes, abbreviation definitions, and consistent decimal-place reporting. Coefficients and standard errors are reported to three decimal places; p-values are reported as exact values or p < .001.

### Supplementary Table S1. Self-developed baseline affective-state items (Manuscript ID: 1901802).

| Item code | Item wording | Response format | Scoring rule | Analytical role |
|---|---|---|---|---|
| BA1 | I feel relaxed at this moment. | 1 = strongly disagree; 7 = strongly agree | Higher scores indicate a more positive baseline affective state. | Pre-exposure relaxation indicator |
| BA2 | I feel stressed at this moment. | 1 = strongly disagree; 7 = strongly agree | Reverse-coded before calculating the baseline affective-state score. | Pre-exposure stress indicator |
| BA3 | I feel mentally fatigued at this moment. | 1 = strongly disagree; 7 = strongly agree | Reverse-coded before calculating the baseline affective-state score. | Pre-exposure mental-fatigue indicator |

*Note.* BA = baseline affective state. The baseline affective-state measure was administered before exposure to the metro-station visual stimuli. BA2 and BA3 were reverse-coded so that higher mean scores indicated a more positive baseline affective state. The mean score of BA1, reversed BA2, and reversed BA3 was used as the baseline affective-state covariate in the serial mediation analysis. The three items were self-developed brief control indicators and were not intended to constitute a clinical or trait affect scale.

### Supplementary Table S2. Covariate predictive effects in the serial mediation model (Manuscript ID: 1901802).

| Outcome variable | Covariate | B | SE | p |
|---|---|---|---|---|
| Perceived crowding | Baseline affective state | −0.135 | 0.046 | .003 |
| Perceived crowding | Age | 0.002 | 0.005 | .766 |
| Perceived crowding | Gender | 0.043 | 0.073 | .558 |
| Perceived crowding | Daily commuting duration | −0.003 | 0.002 | .211 |
| Perceived crowding | Design-related background | 0.035 | 0.112 | .757 |
| Environmental comfort | Baseline affective state | 0.104 | 0.045 | .021 |
| Environmental comfort | Age | 0.001 | 0.005 | .906 |
| Environmental comfort | Gender | 0.015 | 0.070 | .826 |
| Environmental comfort | Daily commuting duration | −0.002 | 0.002 | .524 |
| Environmental comfort | Design-related background | 0.001 | 0.108 | .990 |
| Momentary affective well-being | Baseline affective state | 0.209 | 0.045 | < .001 |
| Momentary affective well-being | Age | −0.003 | 0.005 | .505 |
| Momentary affective well-being | Gender | 0.082 | 0.070 | .244 |
| Momentary affective well-being | Daily commuting duration | 0.003 | 0.002 | .188 |
| Momentary affective well-being | Design-related background | 0.068 | 0.108 | .527 |

*Note.* B = unstandardized regression coefficient; SE = standard error. p-values are reported as exact values or p < .001. The serial mediation model included baseline affective state, age, gender, daily commuting duration, and design-related background as covariates. Categorical covariate coding follows the coding used in the main analysis dataset.

**Supplementary Table S3. Exploratory subgroup mediation results by design-related background (Manuscript ID: 1901802).**

| Indirect pathway | Non-design group effect [95% CI] | Design-related group effect [95% CI] | Difference [95% CI] |
|---|---|---|---|
| Condition → perceived crowding → affective well-being | 0.136 [0.072, 0.215] | 0.233 [−0.010, 0.559] | 0.097 [−0.157, 0.426] |
| Condition → environmental comfort → affective well-being | 0.092 [0.034, 0.160] | −0.022 [−0.337, 0.222] | −0.114 [−0.439, 0.136] |
| Condition → perceived crowding → environmental comfort → affective well-being | 0.034 [0.014, 0.060] | −0.005 [−0.116, 0.054] | −0.039 [−0.157, 0.027] |
| Total indirect effect | 0.261 [0.171, 0.361] | 0.205 [−0.177, 0.538] | −0.056 [−0.454, 0.290] |

*Note.* CI = confidence interval. All confidence intervals are 95% bootstrap confidence intervals. The subgroup comparison is exploratory only and should not be interpreted as a confirmatory moderation test. Because the design-related subgroup was relatively small and all corresponding confidence intervals included zero, the subgroup results should be interpreted cautiously and should not be generalized to broader populations.

## General abbreviation note.

BA = baseline affective state; B = unstandardized regression coefficient; SE = standard error; CI = confidence interval; HSV = hue, saturation, value; CR = composite reliability; AVE = average variance extracted; CFI = comparative fit index; TLI = Tucker-Lewis index; RMSEA = root mean square error of approximation; SRMR = standardized root mean square residual; LLCI = lower limit confidence interval; ULCI = upper limit confidence interval; Boot SE = bootstrap standard error.
